# Genetic diversity and genome-wide association analysis of pine wood nematode populations in different regions of China

**DOI:** 10.3389/fpls.2023.1183772

**Published:** 2023-06-23

**Authors:** Yang Aixia, Ding Xiaolei, Feng Yuan, Zhao Ruiwen, Ye Jianren

**Affiliations:** Co-Innovation Center for Sustainable Forestry in Southern China, College of Forestry, Nanjing Forestry University, Nanjing, China

**Keywords:** *Bursaphelenchus xylophilus*, SNP, genetic diversity, genome-wide association study, temperature tolerance

## Abstract

**Introduction:**

Pine wilt disease (*Bursaphelenchus xylophilus*) was recently detected in Liaoning Province, which was previously considered an unfavourable area for *B. xylophilus* due to its low temperatures. This study aims to compare the reproductivity and genetic variations of *B. xylophilus* isolates from Liaoning Province and other parts of China to explore their phenotypic and genomic differences.

**Methods:**

The samples from Liaoning, Anhui, Hubei, Henan, Zhejiang and Jiangsu were isolated and purified to obtain the strains. The reproductivity of the strains was determined at 15 °C. The genetic structure was analyzed by using SNP molecular markers, and the whole genome association analysis was carried out by integrating SNP information and feculence traits.

**Results:**

A reproductivity experiment showed that Liaoning isolates have higher reproductive ability at 15 °C. Subsequent SNP profiling and population differentiation analysis revealed obvious genetic differentiation of Liaoning isolates from other isolates. A genome-wide association study showed that SNPs closely related to low-temperature tolerance were mainly located in GPCR, Acyl-CoA, and Cpn10, which are responsible for adaptation to environmental factors, such as temperature change.

**Discussion:**

Pine wood nematodes likely adapted to the climate in Liaoning and maintained a certain reproductive capacity at low temperature via variants of adaptation-related genes. This study provides a theoretical basis for elucidating the prevalence and diffusion status of *B. xylophilus* in China.

## 1 Introduction

Pine wilt disease (PWD) is a worldwide forest disease that causes devastating mortality of pine trees. It is currently distributed in 8 countries, namely, the United States, Canada, Mexico, Japan, China, South Korea, Portugal and Spain (Ding et al., 2016; Li et al., 2022a). China is considered one of the countries with the most severe epidemic situation caused by PWD (Ye, 2019). The disease was first discovered in Nanjing, Jiangsu Province, in 1982 and has now spread to 731 county-level administrative regions in 19 provinces (National Forestry and Grassland Administration No. 6 announcement of 2022). As an exotic invasive species, *B. xylophilus* (PWN) is mainly transmitted by human activities such as epidemic wood transport and infrastructure construction (Futai, 2013). Studies have shown that PWNs, the causative agent of PWD, have the ability to resist both high and low temperatures (Wang et al., 2012; Wang et al., 2016; Chen et al., 2021). When the PWN enters a new temperature zone, it can mobilize its phenotypic plasticity to adapt to the local climate and express stable heritable variation (William, 2014; Pan et al., 2020; Rahim et al., 2021). Consequently, the PWN could survive in various environments and spread to many parts of China within decades. In recent years, the PWD has shown a tendency to expand to northern China (Li et al., 2020), which poses a great threat to large pine forests in the area. From 2016 to 2021, PWD was observed in Dalian City and Dandong City of Liaoning Province, as well as Tonghua City and Yanbian Korean Autonomous Prefecture of Jilin Province. This northwards expansion phenomenon indicated that the PWN population has gradually adapted to low-temperature environments over the course of nearly 40 years since its first introduction to China (Li and Zhang, 2018; Zheng et al., 2021).

In the process of adapting to new environments, PWNs undergo founder effects and genetic drift, thus developing a high level of genetic variation (Wang et al., 2022). With the announcement of the genome of the PWN, researchers began to study the genetic diversity among different PWN populations to provide new insights into this devastating disease (Kikuchi et al., 2011; Valadas et al., 2012; Figueiredo et al., 2013; Palomares-Rius et al., 2015; Huang et al., 2019; Ding et al., 2022; Wang et al., 2022). The study of Figueiredo et al., 2013 firstly used single nucleotide polymorphism (SNP) labelling technology to analyse the molecular differences in 7 PWN populations from Portugal, China, the United States and Japan and found that the Portuguese isolates were most similar to those from China, and least similar to the United States and Japanese isolates (Figueiredo et al., 2013). Several studies suggest that genetic diversity is closely related to ecological traits such as pathogenicity, reproductivity and environmental adaptability (Ding et al., 2016; Kong et al., 2021). Recent studies have shown that population clustering is highly correlated with temperature and precipitation (Ding et al., 2022).

In terms of studies on the low-temperature tolerance and adaptability of the PWN population, researchers mainly analysed the low-temperature resistance genes of PWNs based on RNA sequencing. A series of genes were found, including the G protein coupled receptors (BxGPCR), the autophagy marker gene (BxATG8-і and BxATG8-ii), trehalose-6-phosphate synthase encoding gene (Bx-tps), trehalose-6-phosphate phosphatase encoding gene (Bx-tpp) and trehalase encoding genes (Bx-tres) (Chen et al., 2020; Chen et al., 2021), and proved to enable the PWN to deal with temperature changes by participating in signal transduction and regulating autophagy and longevity-related metabolic processes. However, few reports have illuminated the relationship between genome-level variations, such as SNPs, and low-temperature resistance genes among different PWN populations. In contrast, numerous studies have proven that SNP variations are closely associated with many complex features of mammals, plants, and microorganisms, including temperature resistance (Zhu et al., 2008; Ghasemi et al., 2019; Godina et al., 2022; Gomez-Espejo et al., 2022).

Liaoning is known as one of the epidemic areas in China where the annual minimum temperature is below -10°C and the local annual average temperature is lower than 10°C. The annual average temperatures in Anhui, Henan, Hubei, Jiangsu and Zhejiang Provinces are all higher than 10°C, and the regions of Jiangsu and Anhui are among the major distribution areas of PWD in China. The annual average temperatures of these provinces are significantly higher than those in Liaoning Province. The introduction of PWN into Northeast China requires an adaptation process, which is mainly due to the effective accumulated temperature and the minimum temperature in winter in the disaster inducing environment in the middle temperate zone (Jiang et al., 2022). To identify the potential adaptation mechanism of PWN populations, the reproduction and SNP variations of PWNs from Liaoning and southern China were compared. The findings provide a new perspective for revealing the adaptation of PWNs to various environmental factors, such as low-temperature stress.

## 2 Materials and methods

### 2.1 Isolation and purification of nematodes

Fifteen PWN isolates were collected from infected trees located in Liaoning, Anhui, Hubei, Henan, Jiangsu and Zhejiang Provinces. All isolates were isolated from felled trees by the Baermann funnel method (Viglierchio and Schmitt, 1983). After preliminary identification based on the morphological characteristics of PWNs (Xie, 2005), molecular identification was performed to ensure detection accuracy. Approximately 15 male and female individuals were selected from one tree and cultured on a colony of *Botrytis cinerea* fungus at 28 °C. After cultivation, the nematodes were separated by the Baermann funnel method, washed with 0.05% streptomycin sulfate and sterile water, and stored in a refrigerator at 4 °C for later use and subcultured every 5-6 months.

A total of 15 *B. xylophilus* isolates were collected, including 5 from Liaoning (LN12, LN15, LN16, LN17, and LN18), 2 from Anhui (AH03 and AH32), 2 from Hubei (HB06 and HB10), 2 from Henan (HEN09 and HEN17), 2 from Jiangsu (JS08 and JS20), and 2 from Zhejiang (ZJ27 and ZJ29) (**Table 1** and **Supplementary Figure S1**).

**Table 1:** Sample information of 15 *B. xylophilus* isolates.

Strain No.	Origin	Average temp/°C	Annual rainfall/mm	Host	Sampling date
LN12	Kaiyuan, Tieling City, Liaoning Province	3-15	678	*Pinus tabuliformis*	2017.11
LN15	Qingyuan County, Fushun City, Liaoning Province	0-14	804.2	*Larix gmelinii*	2018.09
LN16	Xinbin City, Fushun City, Liaoning Province	0-14	804.2	*L. gmelinii*	2018.09
LN17	Xinbin City, Fushun City, Liaoning Province	0-14	804.2	*L. gmelinii*	2018.09
LN18	Fushun City, Liaoning Province	0-14	804.2	*L. gmelinii*	2018.09
AH03	Quanjiao County, Chuzhou City, Anhui Province	13-22	1031.2	*Pinus massoniana*	2015.03
AH32	Huangshan District, Huangshan City, Anhui Province	13-22	1670	*P. massoniana*	2018.10
HB06	Huangpi District, Wuhan City, Hubei Province	13-22	1200	*P. massoniana*	2015.04
HB10	Yidu District, Yichang City, Hubei Province	14-22	1350	*P. massoniana*	2015.11
HEN09	Xichuan County, Nanyang City, Henan Province	12-22	800	*P. massoniana*	2017.11
HEN17	Xixia County, Nanyang City, Henan Province	12-22	800	*P. massoniana*	2018.10
JS08	Yixing City, Wuxi City, Jiangsu Province	13-22	1079.3	*P. massoniana*	2014.12
JS20	Changshu, Suzhou City, Jiangsu Province	14-22	1094	*P. massoniana*	2017.10
ZJ27	Tonglu County, Hangzhou City, Zhejiang Province	14-23	1378.5	*P. massoniana*	2014.08
ZJ29	Tiantai County, Taizhou, Zhejiang Province	14-23	1242.5	*P. massoniana*	2015.07

### 2.2 Determination of *B. xylophilus* reproduction at 15 °C

We had a literature survey on the PWN population dynamics and temperature changes along the year in Liaoning and other provinces. Based on the available information, we found 15 °C as an appropriate low temperature threshold to compare reproductivity differences between 5 PWN isolates from Liaoning and other 10 nematode isolates from other regions (Lu, 2015; Kong et al., 2021; Zheng et al., 2021).

For inoculation, the number of each nematode isolate was manually adjusted to 100 (the ratio of female to male was close to 1-1) in 20 µl of sterile water. One hundred individuals were inoculated onto *B. cinerea* mycelial mats maintained on potato dextrose agar (PDA) in 7 cm Petri dishes at 15°C for 5 days with three replicates. The feeding condition of nematodes was observed every day. After culture for 5 days, the nematodes were extracted by the Baermann funnel method and counted. Analysis was performed using RStudio (https://www.rstudio.com/).

### 2.3 Genome resequencing

The DNA extraction and sequencing for individual isolate was performed immediately after sampling. DNA was extracted from the PWNs using the CTAB method (Zhang et al., 2005). The extracted DNA was stored in the PWN DNA repository of the Laboratory of Forest Pathology, Nanjing Forestry University. DNA concentration and quality were assessed by a NanoDrop Qubit instrument (Thermo Fisher) and visualization in agarose gel. The qualified DNA was sent to Wuhan Future Group Biological Company for high-throughput genome sequencing on an Illumina HiSeq 4000 platform. The genome resequencing method was 150 bp paired-end sequencing, and the average sequencing depth was greater than 40×.

### 2.4 Identification and filtration of mutation sites

The quality of the raw sequencing data was first assessed by FastQC (http://www.bioinformatics.babraham.ac.uk/projects/fastqc/). Filtered reads were aligned to the PWN reference genome announced in 2021 (Ding et al., 2022) by BWA (http://bio-BWA.SourceForge.net/BWA.shtml). SAMtools (http://samtools.sourceforge.net/samtools.shtml) and Picard (http://broadinstitute.github.io/picard/) were used to remove duplicates. Putative SNPs were called by FreeBayes (https://github.com/ekg/freebayes) with a minimum coverage > 10, and VCFtools (https://github.com/vcftools) was used for SNP site statistical analysis.

### 2.5 Genetic differentiation and genome-wide association analysis

The SNPs with low allele frequency, high linkage disequilibrium and missing rate were filtered by the snpgdsLDpruning function in SNPRelate package (https://www.bioconductor.org/packages/release/bioc/html/SNPRelate.html) of RStudio software (https://www.rstudio.com/). A principal component analysis (PCA) was performed using the filtered SNPs *via* snpgdsPCA function in the same SNPRelate package and the visualized by the ggplot2 package (https://cran.r-project.org/web/packages/ggplot2/). PLINK (v1.9) (https://www.cog-genomics.org/plink/) was used to extract the filtered site information to generate new vcf file for phylogenetic tree analysis. VCF-kit (https://vcf-kit.readthedocs.io/en/latest/) and MEGA (v11.0.11) (https://www.megasoftware.net/) was used to construct phylo-tree using the neighbour-joining method.

According to the phenotypic data obtained in 2.2, all isolates were divided into two groups: Liaoning and other isolates for genome-wide association analysis. Firstly, PLINK was used to obtain the corrected p value for each SNP site based on group information. Then, the qqman package (https://cran.r-project.org/web/packages/qqman/) was used to create Manhattan plots for genome-wide association study (GWAS) data from the Plink results. The SNP loci significantly related to low-temperature resistance were annotated using the annotate_variator.pl script in ANNOVAR software (v2018Apr16) (Wang et al., 2010). Significant SNPs caused by host and other factors were excluded according to the annotation results. Gene Ontology (GO) enrichment analysis was performed using the topGO package (https://rdrr.io/bioc/topGO/). Finally, the sequences of the identified SNPs were verified by Sanger sequencing.

## 3 Results

### 3.1 Comparison of the reproductivity of different isolates of *B. xylophilus* at 15 °C

**Figure 1:**
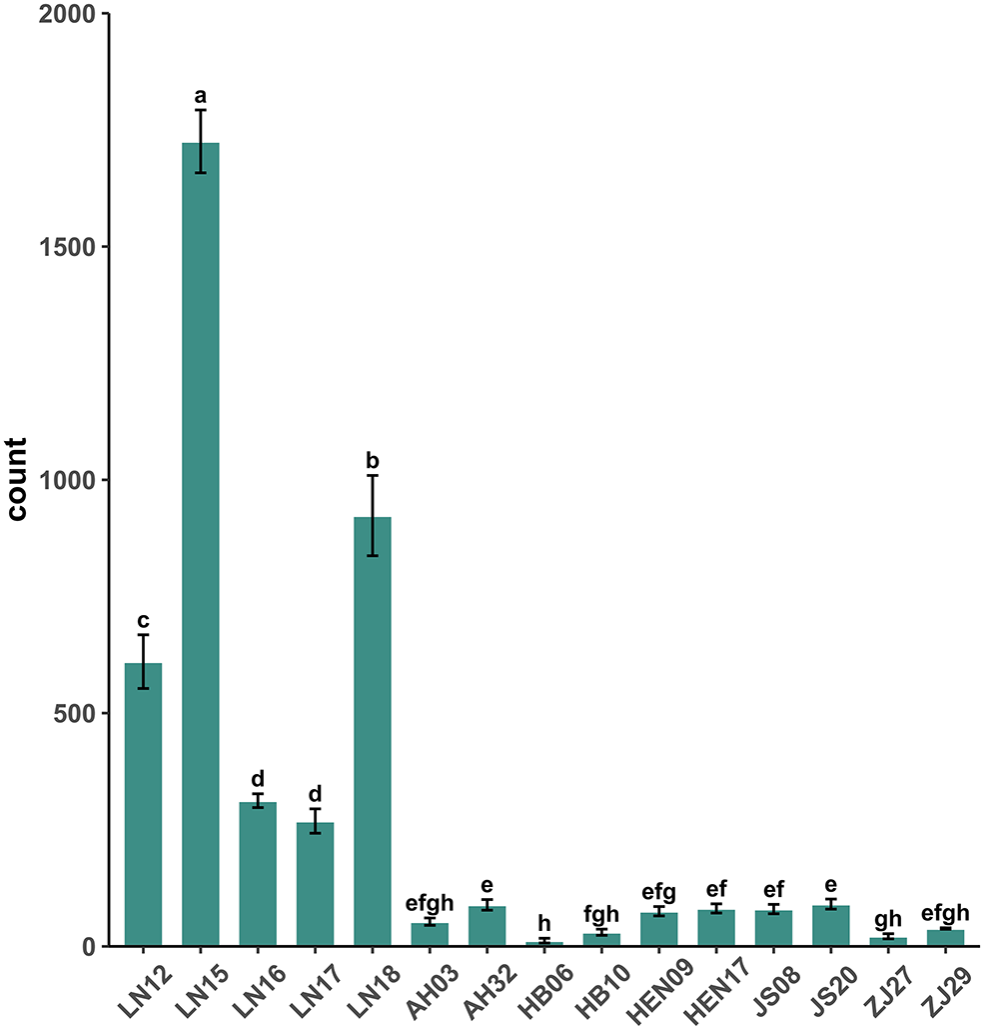
Reproductivity of each strain at 15 °C. Different letters in the figure indicate significant differences (significance level p < 0.05).

Calculation of the reproductivity of different *B. xylophilus* isolates indicated that LN15 showed the strongest reproduction capacity at 15°C, while HB06 exhibited the lowest capacity. Generally, the reproductivity of Liaoning isolates was significantly higher than those from other areas. Within the Liaoning population, LN17 showed the weakest reproductivity, with a mean number of nematodes of only 269 after 5 days. Among other populations, the reproductivity of all isolates was lower at 15 °C. JS20 had the highest reproductivity, with only 91 nematodes observed; however, there were no significant differences among the 10 isolates (**Figure 1**). These results suggest that Liaoning isolates have stronger tolerance than other isolates to low temperature.

### 3.2 Statistics of SNP genotypes and loci

**Figure 2:**
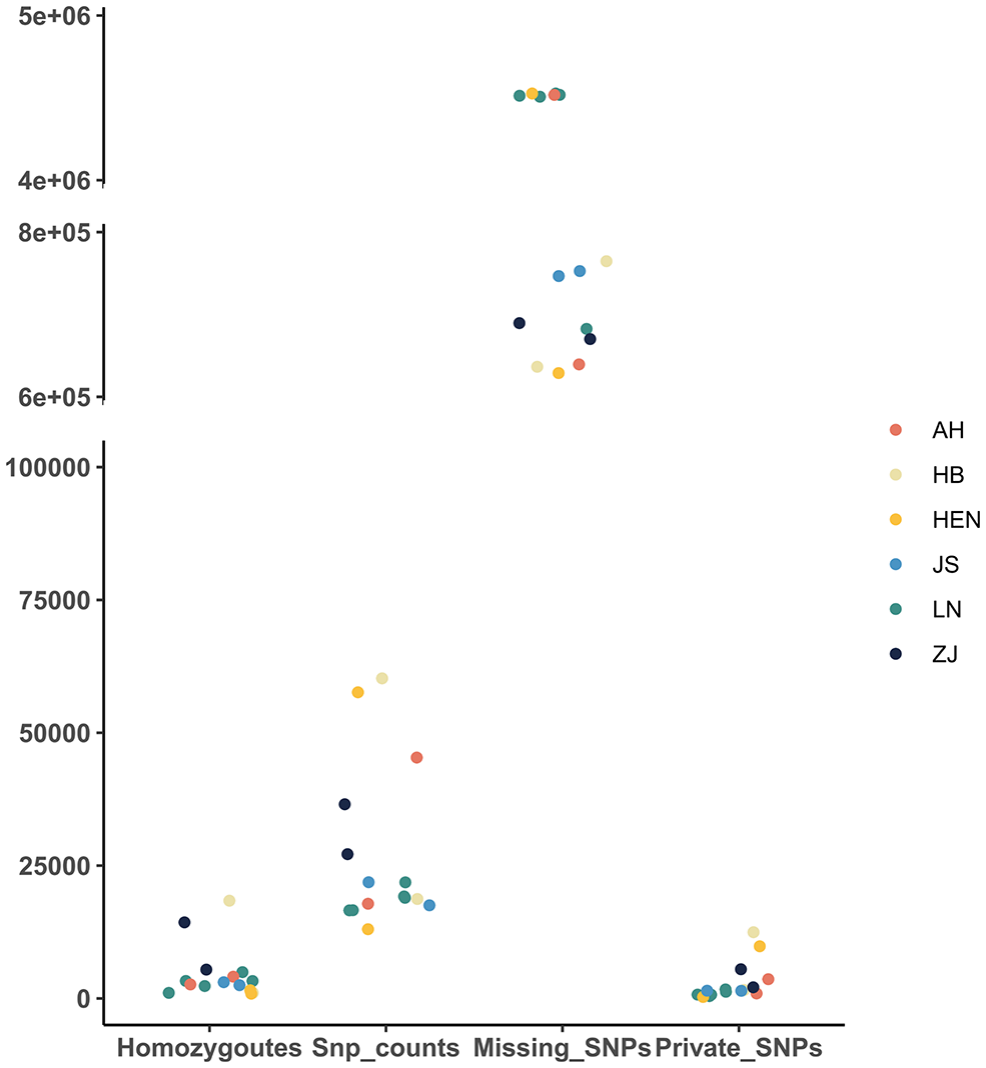
Homozygosity, SNP count, missing SNP and private SNP distributions of 15 isolates.

**Figure 3:**
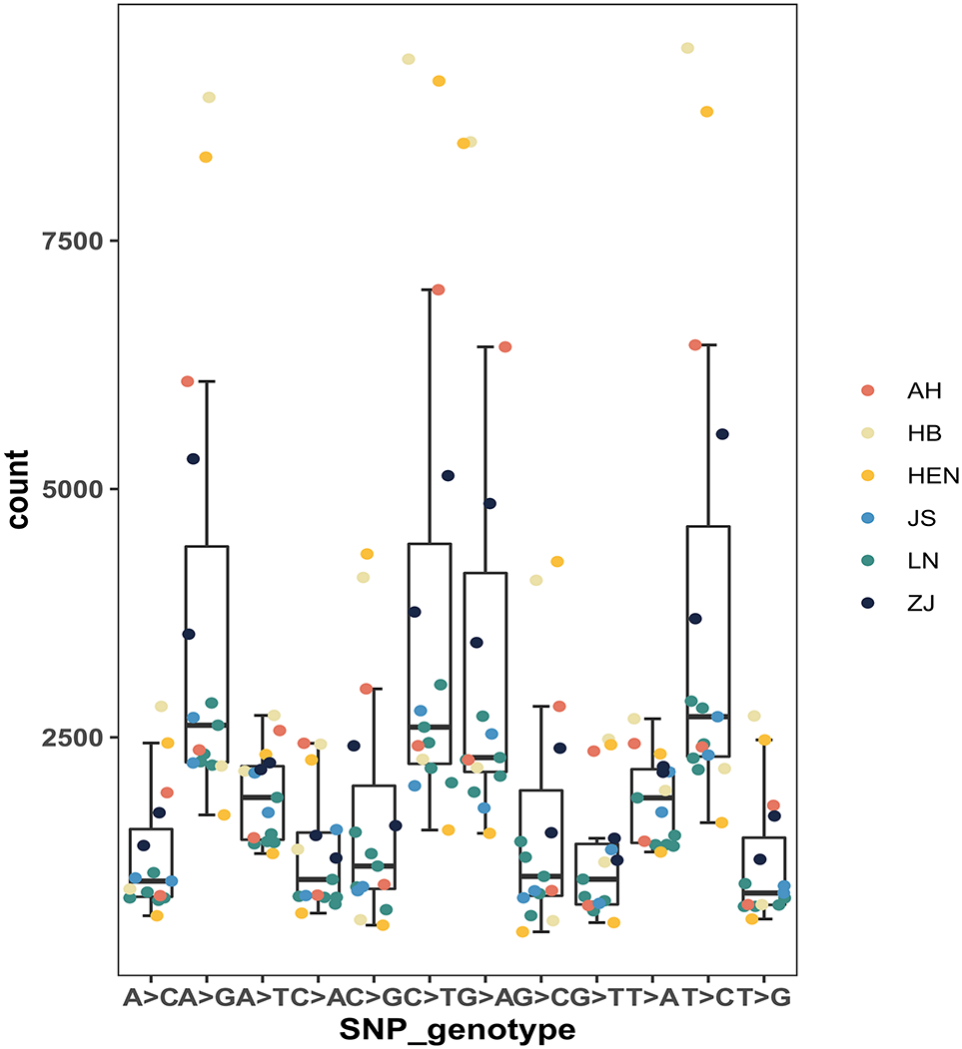
Box plots of SNP genotypes among 15 *B xylophilus* isolates. AH refers to Anhui isolates, HB to Hubei isolates, HEN to Henan isolates, JS to Jiangsu isolates, LN to Liaoning isolates, and ZJ to Zhejiang isolates. The same is shown below.

A total of 121486 SNPs were obtained from 15 PWN isolates, and the number of SNPs in each nematode isolate varied significantly (**Table 2**; **Figure 2**). HB10 possessed most SNPs, homozygous SNPs and private SNPs, while HEN17 possessed the fewest. The largest number of missing SNPs was observed for HEN17 (4526342), and the smallest number was observed for HEN09 (628816). The mean numbers of SNPs and private SNPs in Liaoning isolates were lower than those from other provinces, where the mean value of missing SNPs was higher than that in other provinces. The number of missing SNPs in LN15, LN16, LN17, LN18, AH32 and HEN17 was significantly different from that in other isolates. In addition, there were 12 genotypes among all isolates. In general, the frequencies of the 4 genotypes A>G, C>T, G>A and T>C were significantly higher than those of other genotypes (**Figure 3**).

**Table 2:** A summary of the SNPs found in 15 *B. xylophilus* isolates.

Strain No.	SNP		Homozygous		Missing		Private	
	count	mean	count	mean	count	mean	count	mean
LN12	21,850	18,633	4,953	2,982	682,558	3,749,117	705	964
LN15	16,576		3,293		4,518,034		441	
LN16	19,200		3,278		4,512,384		1,253	
LN17	18,936		2,336		4,506,607		1700	
LN18	16,602		1,051		4,526,004		720	
AH03	45,335	31,580	4,100	3,369	639,384	2,578,540	3,627	2,284
AH32	17,825		2,637		4,517,696		940	
HB06	18,718	39,481	1,049	9,722	764,697	700,580	1,588	7,026
HB10	60,243		18,395		636,462		12,464	
HEN09	57,621	35,325	1,537	1,219	628,816	2,577,579	9,820	5,050
HEN17	13,028		900		4,526,342		280	
JS08	21,874	19,701	3,055	2,778	746,687	749,712	1,459	1,448
JS20	17,528		2,501		752,736		1,437	
ZJ27	27,155	31,850	5,427	9,873	670,134	679,823	2,096	3,797
ZJ29	36,545		14,318		689,511		5,497	

### 3.3 Analysis of genetic differentiation among all *B. xylophilus* isolates

**Figure 4:**
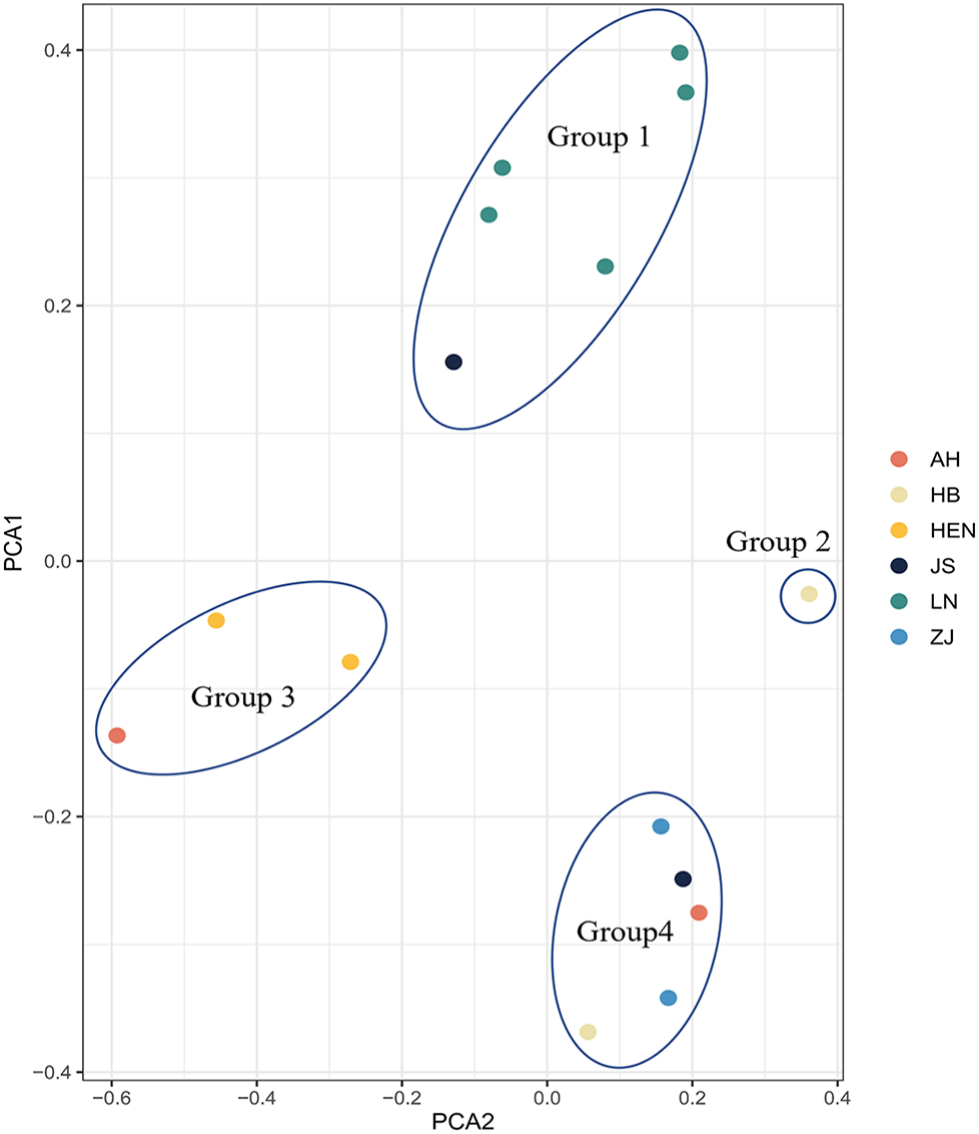
PCA results of the 15 isolates based on 1877 SNP markers.

**Figure 5:**
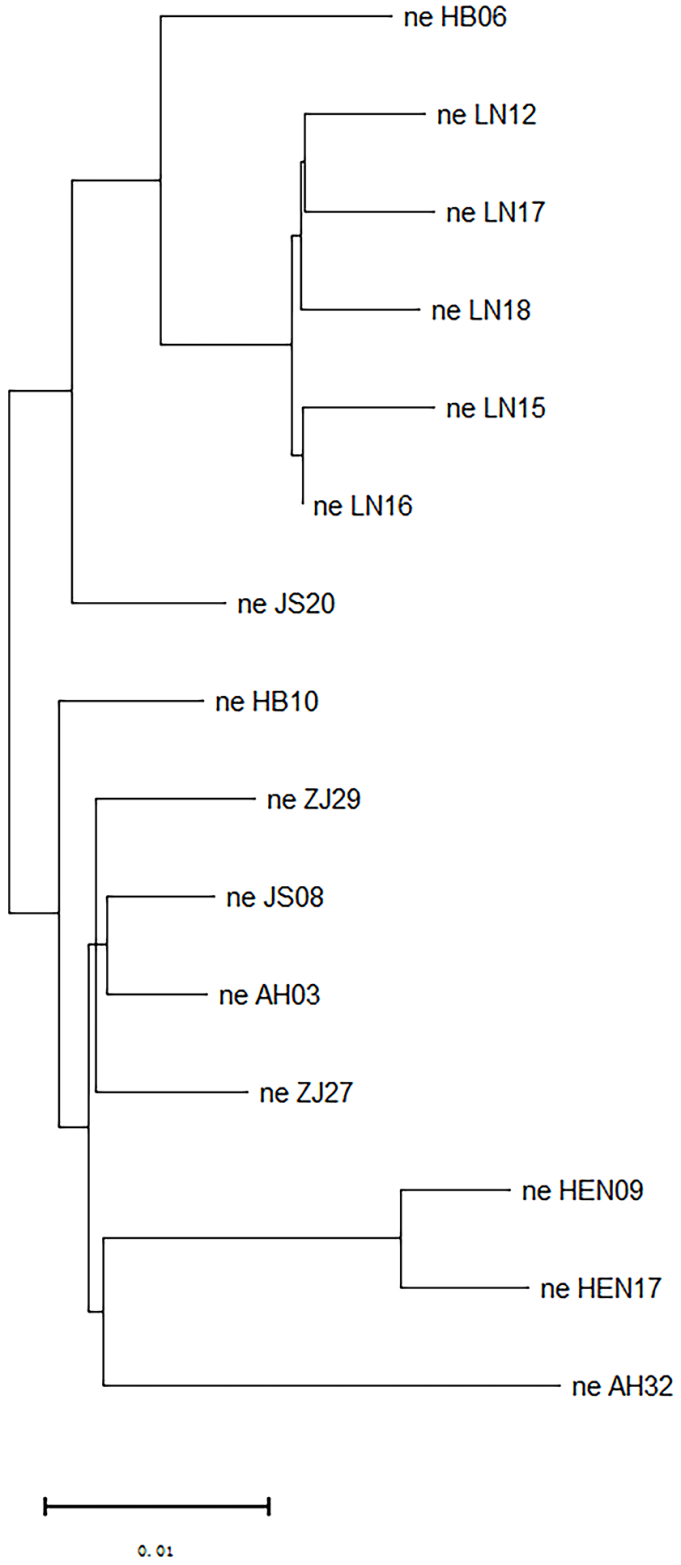
Phylogenetic tree of all 15 isolates constructed by using the neighbour-joining method.

PCA exhibited that the 15 PWN isolates from Liaoning, Anhui, Hubei, Henan, Jiangsu and Zhejiang Provinces could be divided into four groups (**Figure 4**). Group 1 included all Liaoning isolates and one Jiangsu isolate (JS20). Group 2 included only one strain, HB06. Group 3 comprised all Henan isolates and one Anhui strain (AH32). Group 4 contained a total of 5 isolates, including all Zhejiang isolates, one Hubei isolate, one Jiangsu isolate (JS08) and one Anhui isolate (AH03). The above findings were supported by the phylogenetic tree constructed based on SNP loci (**Figure 5**). Isolates from Liaoning showed obviously distinct genetic variations when compared with others.

### 3.4 SNPs highly associated with low-temperature tolerance

**Figure 6:**
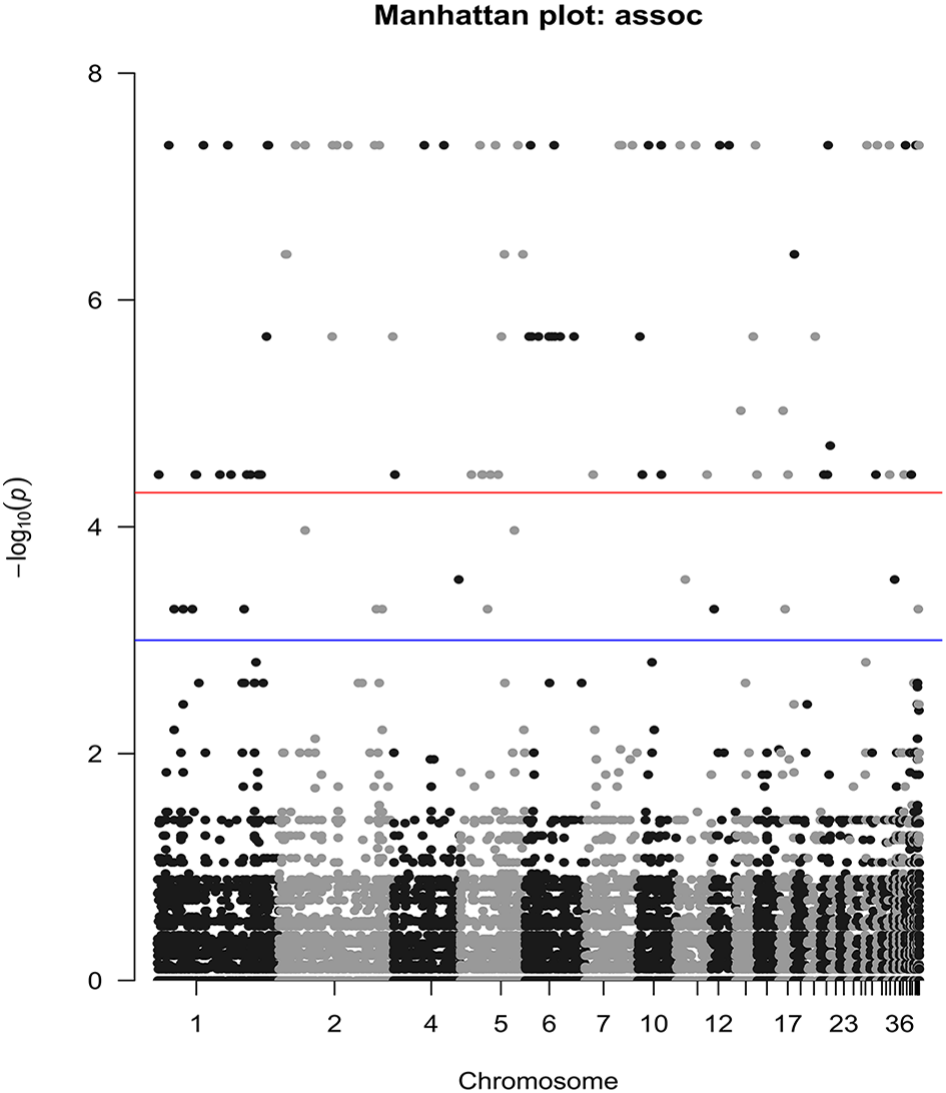
Manhattan plot of SNPs strongly associated with low-temperature tolerance. The blue line is the genome-wide suggestive line = −log10(1e-03); the red line is the genome-wide significance line = −log10(5e-05).

**Figure 7:**
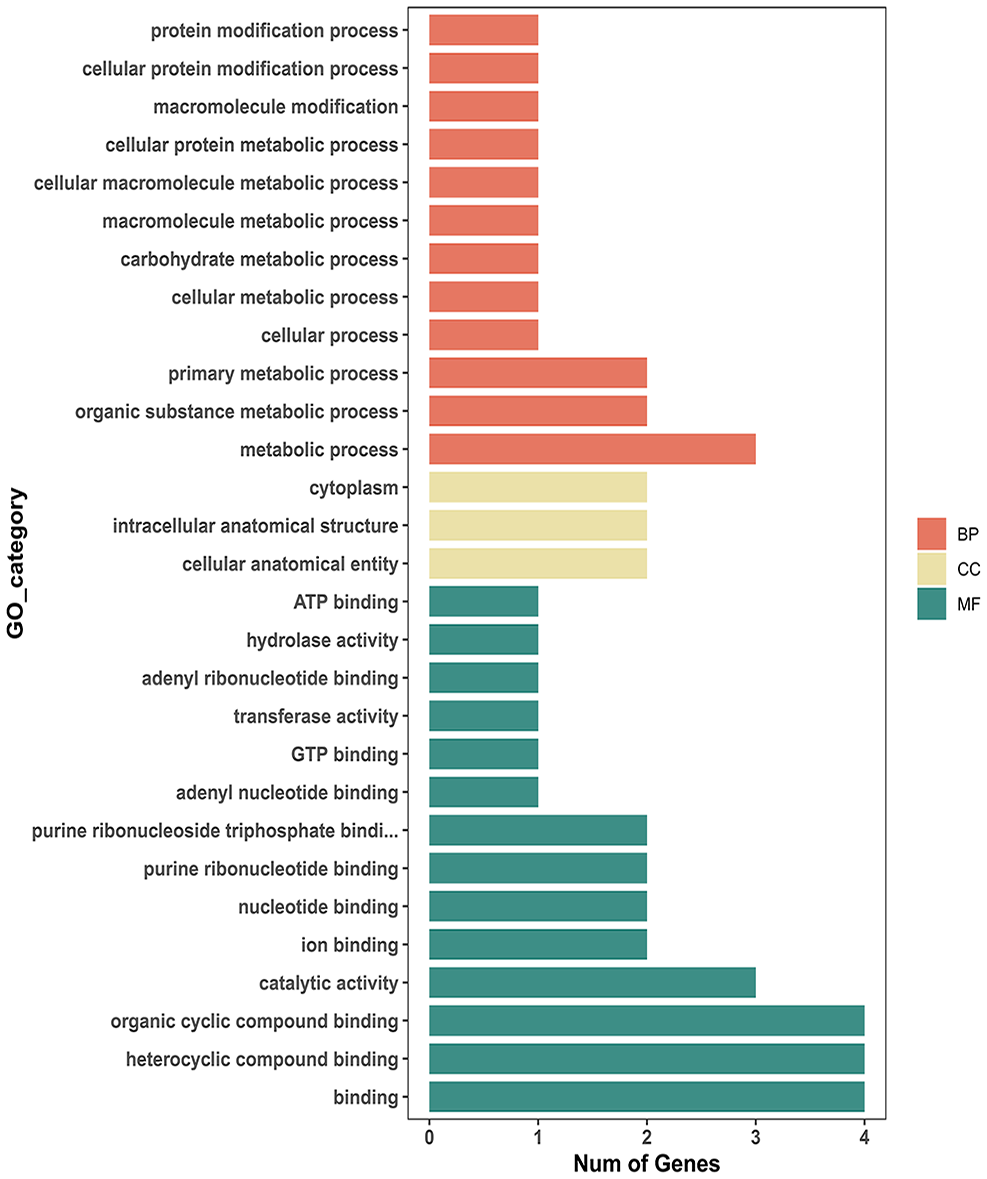
Bar plot of overrepresented GO terms for genes with temperature-associated SNPs (adjusted P value ≤0.05).

The reproductivity and population genetics results suggested that the low-temperature adaptability of Liaoning isolates was different from that of other isolates. Therefore, we searched for SNPs related to tolerance of low temperatures or other environmental stresses in Liaoning isolates using genome-wide association analysis. A total of 277 significantly associated SNPs were detected (P <0.001), 24 of which were located in exons (**Figure 6**). 64% of these exonic SNPs were identified as nonsynonymous SNPs, which will change the protein sequences. Highly associated SNPs (nonsynonymous) were found in the 7TM GPCR (bx1.05233-RA, Contig004:6330970), Acyl-CoA (bx1.12564-RA, Contig245:176) and Chaperonin-Cpn10 (bx1.12514-RA, Contig125:14605) families, which may have a notable correlation with temperature adaptation. The Sanger sequencing result of aforementioned SNPs were conssitent with our genome resequencing data (**Figure S2**). GO enrichment analysis also suggested that the identified SNPs were involved in protein modification or metabolic processes (Cpn10 involved) and GTP binding (GPCR involved). Other proteins, such as ATPase, are related to the regulation of the adaptation and growth of *B. xylophilus via* catalytic activity and metabolic processes (**Figure 7**).

## 4 Discussion

PWD has been spreading to northern China (Huang et al., 2019; Wang et al., 2022) since the first occurrence of PWD in 2016 in Liaoning Province, 7 cities and 19 counties have developed into PWD epidemic areas as of 2022. It is obvious that the PWN has evolved to withstand the low-temperature conditions in the above areas in northern China. It is important to explore potential adaptation mechanisms of the PWN to expand our knowledge of this invasive species. Since SNP molecular markers were first used to study the genetic diversity of PWNs in 2013, several studies have suggested that SNP markers are effective to study the population differentiation of PWNs (Figueiredo et al., 2013). Genome wide association study (GWAS) based on SNP markers is the best way to study the relationship between phenotype and genotype (Godina et al., 2022). Therefore, we tried to compare the genetic diversity between PWNs from low-temperature regions with PWNs from suitable regions, and attempted to explore the low-temperature resistance of PWNs in China by GWAS.

Previous results showed that all PWNs died within a short time at -10°C (Li et al., 2022b). However, PWNs can survive in the cold winter in Liaoning Province, where the annual minimum average temperature is -10°C, and spread continuously (Chen, 2022; Jiang et al., 2022). It is possible that after long-term domestication (since PWNs have been discovered in China for over 40 years) some PWNs in Liaoning Province have evolved to overcome extreme low tempretures (Huang, 2015). Besides, some studies have shown that some PWNs no longer reproduce and a small number of them die at the temperature of 10 °C, but the reproduction of northern and southern PWN isolates displays obvious differentiation at a temperature of 16 °C (Kong et al., 2021). As found in our study, Liaoning isolates had higher reproductivity than isolates from Anhui, Zhejiang, Hubei, Henan and Jiangsu at 15 °C. This indicated that PWNs from Liaoning acquired certain environmental adaptation variation during the colonization process (Wang et al., 2019; Pan et al., 2020; Godina et al., 2022). Meanwhile, the subsequent SNP analysis also indicated the significant genetic changes occurred in temperature associated genes like GPCR, Acyl-CoA and Cpn10.

The SNP locus statistics showed that the mean number of SNPs, private SNPs and missing SNPs of the Liaoning population were significantly different from those of other populations. This is consistent with the following clustering result. Notably, most Liaoning isolates had large numbers of missing SNPs. The missing SNPs were mainly caused by the significant genetic difference in certain isolate. Based on the PCA results, the HB06 isolate was quite different from the others. Based on our inspections, the missing SNPs found in Liaoning province can be mostly found in HB06 isolate. This unexpected high number of missing SNPs could be caused by the obvious intraspecific differences found in HB06 isolate since those missing SNPs were not associated with any key genes with potential regulatory roles.

In addition, cluster analysis showed that all isolates from Liaoning Province were genetically differentiated from other isolates except one Jiangsu isolate. Therefore, the Liaoning isolates had obvious genetic variations compared with the other isolates. Studies have shown that the PWN has genes and epigenetic modifications that enable it to cope with low temperatures and other harsh environmental conditions (Pan et al., 2020; Rahim et al., 2021). Different environmental stresses can lead to epigenetic modifications at different gene loci. Jiangsu Province, Anhui Province and their surrounding areas are the early endemic areas of the PWN in China. Stimulated by long-term phenotypic plasticity, the PWN has formed stably heritable variations and has fully adapted to local climate conditions. In recent years, PWD has spread to Liaoning Province. Due to the large difference in annual average temperature between the Liaoning and Jiangsu regions, Liaoning isolates may be exposed to a series of regulations during colonization, such as regulations of signal transduction, lipid metabolism, growth and development, and antioxidant production (Zhang, 2017; Wang, 2020).

In this study, the SNPs strongly associated with low-temperature tolerance occurred within genes such as 7TM GPCR family members, acyl-CoA and chaperonin-Cpn10. G proteins can bind the nucleotide guanosine triphosphate (GTP) with guanosine diphosphate (GDP) and mediate downstream signal transduction to participate in extracellular signal transduction. They play an important role in the low-temperature response of *B. xylophilus* (Wang et al., 2019). Studies have also shown that G-protein-coupled receptor (GPCR) is essential for the induction of heat shock responses in *Caenorhabditis elegans* (Moria et al., 2013). Acyl-CoA family members function in fatty acid metabolism. Studies have shown that acs-4, a gene associated with acyl-CoA, is involved in the process of fatty acid metabolism and regulates the diapause of nematodes to improve resistance to unsuitable environmental conditions (Zhang, 2019). Studies have linked changes in the expression of some heat shock protein (HSP) family genes with adaptation to temperature changes in the PWN (Hao et al., 2022). For example, Bx-HSP90 (heat shock protein 90) functions as a temperature regulator, which is essential for the survival of PWNs (Wang et al., 2012). Therefore, we hypothesized that Cpn10 regulates the temperature adaptation of PWNs (Mande et al., 1996). Meanwhile, the GO enrichment results showed that these identified SNPs were enriched in gene functions such as GTP binding and transferase activity, biological processes such as protein modification or metabolic process, and cellular components. GPCR binds the G protein and hydrolyses guanosine 5’-triphosphate (GTP), which can mediate downstream signal transduction and participate in extracellular signal transduction (Moria et al., 2013). Protein-related processes and organic compound binding may be involved in regulation of the HSP family (Wang et al., 2012). This further proves that SNPs may be one of the effective methods to explore the mechanism of low temperature tolerance of PWN. Unfortunately, its protein function was not verified in this study, we believed that more research is needed to functionally or metabolically confirm the validity of above key genes identified *via* GWAS in this study, meanwhile, the influence of other environmental factors must not be discarded.

In conclusion, this study showed that Liaoning isolates had obvious high reproductivity at low temperatures and significant differences in genetic information. According to the GWAS results, we suggest that many of the temperature-associated genes were mutated in Liaoning isolates, which could be responsible for its better survival and reproduction abilities under low temperature. Our study provides a theoretical basis for further understanding the epidemic and diffusion of PWD in China.

## Data availability statement

The original sequencing data have been deposited in the NCBI archive, PRJNA524063. The data that support the findings of this study are openly available in NCBI at https://www.ncbi.nlm.nih.gov/.

## Author contributions

YA: Designed the study, conducted the experiment, performed the data analysis and wrote the article. DX: Guided the article writing and data analysis. FY and ZR: Collected the samples. YJ: Guaranteed the integrity of the entire study and approved the final version of the manuscript. All authors have read and agreed to the published version of the manuscript. All authors contributed to the article and approved the submitted version.

## Supplementary material

Supplementary Figure 1 The collecting locations of 15 *B. xylophilus* isolates.

Supplementary Figure 2 The resequencing of SNPs related to low-temperature tolerance.
