# Supplementary figures and images for: Genetic diversity and genome-wide association analysis of pine wood nematode populations in different regions of China
Source: Front Plant Sci. 2023 Jun 23;14:1183772. doi: 10.3389/fpls.2023.1183772 (PMC10327295; doi:10.3389/fpls.2023.1183772)

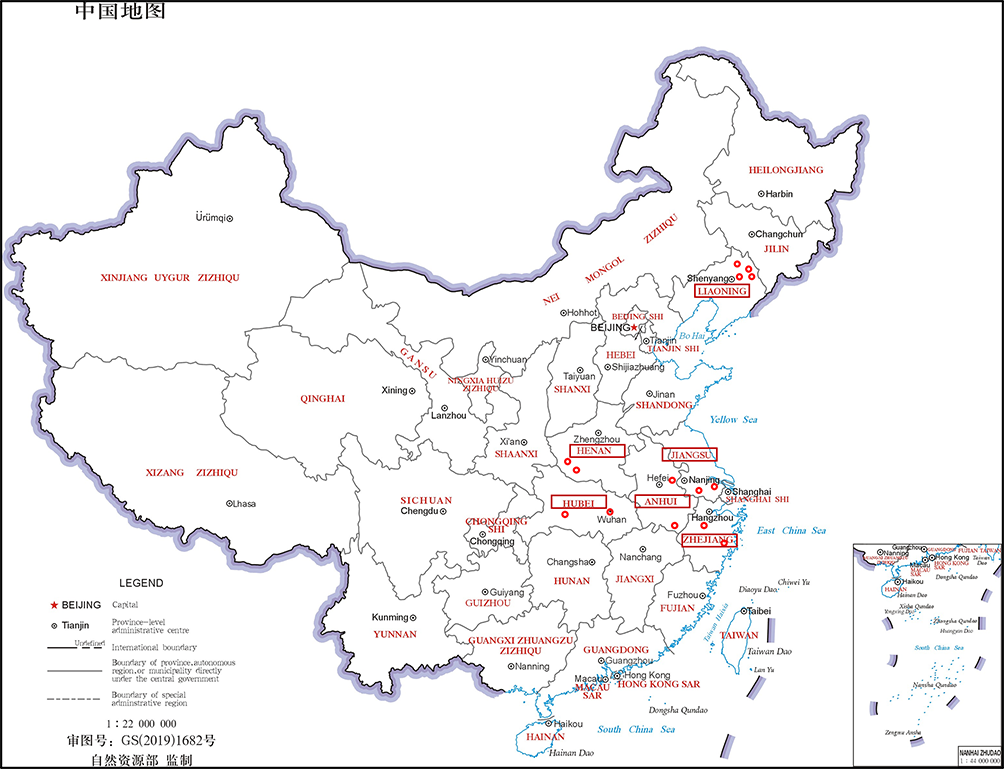

Supplement: Supplementary Figure 1 — The collecting locations of 15 B. xylophilus isolates. [file Image_1.tif]

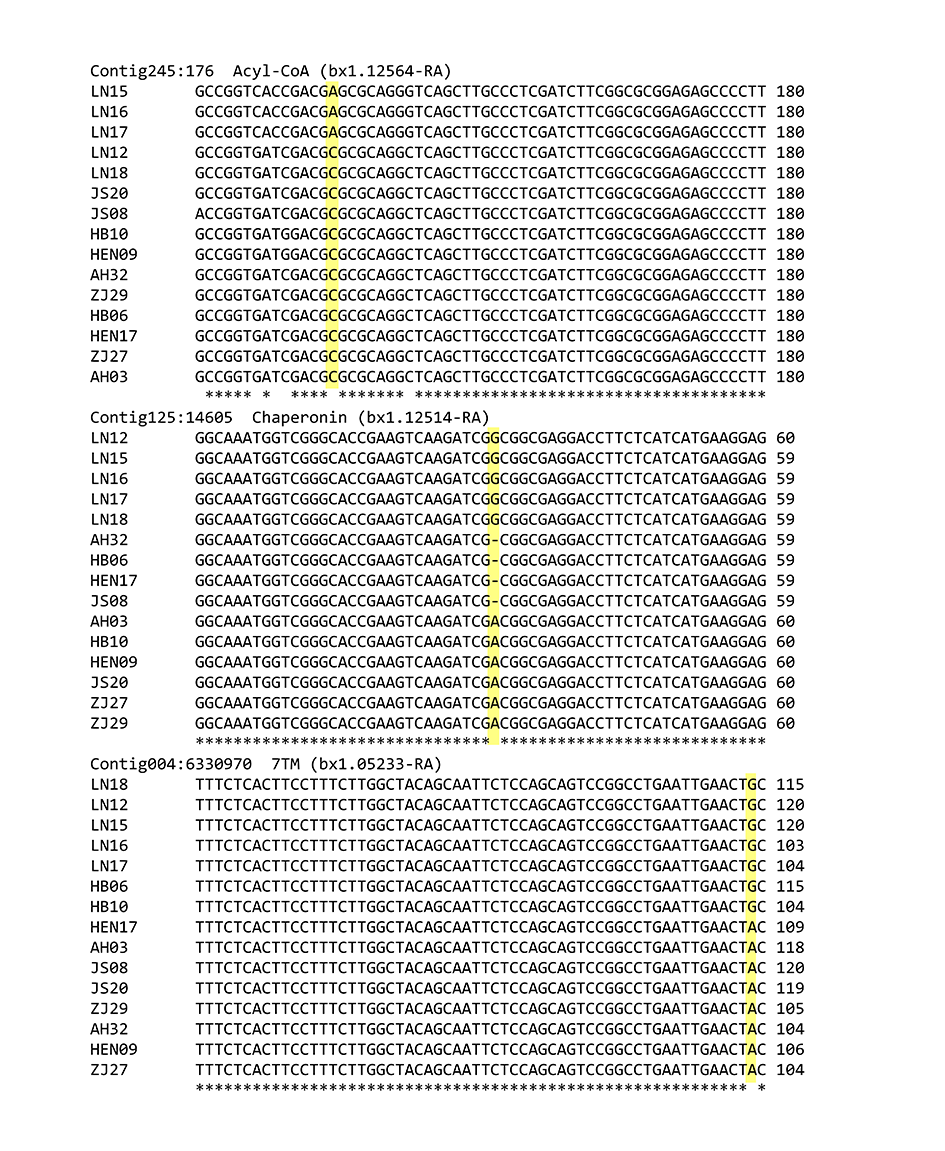

Supplement: Supplementary Figure 2 — The resequencing of SNPs related to low-temperature tolerance. [file Image_2.tif]
